# Implementation of national whole-genome sequencing of *Mycobacterium tuberculosis*, National Public Health Laboratory, Singapore, 2019—2022

**DOI:** 10.1099/mgen.0.001139

**Published:** 2023-11-27

**Authors:** Ansel Yi Herh Lim, Michelle L.T. Ang, Sharol S. L. Cho, Deborah H. L. Ng, Jeffery Cutter, Raymond T. P. Lin

**Affiliations:** ^1^​ National Public Health Laboratory, National Centre for Infectious Diseases, Singapore, Singapore; ^2^​ National Tuberculosis Programme, National Centre for Infectious Diseases, Singapore, Singapore

**Keywords:** drug susceptibility testing, outbreak investigation, public health microbiology, tuberculosis surveillance, whole-genome sequencing

## Abstract

The National Tuberculosis Programme (NTBP) monitors the occurrence and spread of tuberculosis (TB) and multidrug-resistant TB (MDR-TB) in Singapore. Since 2020, whole-genome sequencing (WGS) of *Mycobacterium tuberculosis* isolates has been performed at the National Public Health Laboratory (NPHL) for genomic surveillance, replacing spoligotyping and mycobacterial interspersed repetitive unit-variable number tandem repeats analysis (MIRU-VNTR). Four thousand three hundred and seven samples were sequenced from 2014 to January 2023, initially as research projects and later developed into a comprehensive public health surveillance programme. Currently, all newly diagnosed culture-positive cases of TB in Singapore are prospectively sent for WGS, which is used to perform lineage classification, predict drug resistance profiles and infer genetic relationships between TB isolates. This paper describes NPHL’s operational and technical experiences with implementing the WGS service in an urban TB-endemic setting, focusing on cluster detection and genomic drug susceptibility testing (DST). Cluster detection: WGS has been used to guide contact tracing by detecting clusters and discovering unknown transmission networks. Examples have been clusters in a daycare centre, housing apartment blocks and a horse-racing betting centre. Genomic DST: genomic DST prediction (gDST) identifies mutations in core genes known to be associated with TB drug resistance catalogued in the TBProfiler drug resistance mutation database. Mutations are reported with confidence scores according to a standardized approach referencing NPHL’s internal gDST confidence database, which is adapted from the World Health Organization (WHO) TB drug mutation catalogue. Phenotypic–genomic concordance was observed for the first-line drugs ranging from 2959/2998 (98.7 %) (ethambutol) to 2983/2996 (99.6 %) (rifampicin). Aspects of internal database management, reporting standards and caveats in results interpretation are discussed.

## Data Summary

This publication analyses the lineage distribution and genotypic–phenotypic drug susceptibility correlation data derived from tuberculosis whole-genome sequencing performed in our laboratory. These datasets are made available in the Supplementary Material, available in the online version of this article.

**Impact Statement**

This paper describes the operational workflow and technical methods used in Singapore’s national whole-genome sequencing (WGS) programme, which has been developed gradually over the past decade. By sharing NPHL’s unique experiences and challenges in WGS implementation, the paper adds to the currently scarce literature on the public health laboratory implementation of WGS in urban endemic settings with moderate to high incidence rates. We have demonstrated the feasibility of a universal prospective approach to WGS, and shown its utility in targeting contact screening, justifying mass screening of residential apartment blocks, and discovering hidden social networks. Our laboratory’s implementation experiences may serve as a useful reference for public health laboratories planning to set up WGS services.

## Background of tuberculosis surveillance in Singapore

Singapore is a city-state with a high population density. In the 1960s, the crude incidence rate of tuberculosis (TB) disease was over 300 cases per 100 000 persons. By the mid-1980s, incidence rates had declined to approximately 50 cases per 100 000 population. Short-course chemotherapy for TB disease was introduced in 1985. The Singapore TB Elimination Programme (STEP) was initiated in 1997, with the subsequent rollout of directly observed short-course therapy. Since the mid-2000s, incidence rates have remained between 30 to 40 cases per 100 000 people, with the ageing local population and influx of migrants from high-incidence countries being contributing factors to the non-decline [1]. In 2018, the prevalence of latent TB (TB infection) in Singapore residents was estimated at 12.7 % based on QuantiFERON interferon gamma release assay (IGRA) in a cross-sectional study of 1682 Singapore residents [2], with a prevalence of 29.4 % among persons aged 70–79 years old. Latent TB prevalence may be higher in the migrant population, with overall prevalence estimated to be 20.4 % by a QuantiFERON-based cross-sectional study of 3584 migrants [3].

Like other globalized cities with migrant populations, Singapore is exposed to the threat of multidrug-resistant tuberculosis (MDR-TB). Among persons born in Singapore or Malaysia who were diagnosed with culture-positive pulmonary TB between 2002 and 2014, the incidence of MDR-TB was 0.3 % [4]. There is a risk of spread of MDR-TB within and across Singapore’s national borders. Local spread of MDR-TB between migrants and residents had previously been reported in a correctional setting [5], and a local adolescent with no travel or contact history was diagnosed with MDR-TB in 2011 [6]. There have also been reports of MDR-TB clusters related to two gaming centres in 2012 [7] and an 11-storey apartment block between 2012 and 2016 [8].

## Role of the national public health laboratory in tuberculosis surveillance

Spacer oligonucleotide typing (spoligotyping) and 24-loci mycobacterial interspersed repetitive unit-variable number of tandem repeats (MIRU-VNTR) are low to mid-resolution molecular genotyping methods that are well established for TB surveillance [9]. These methods were previously used by STEP to assign TB lineages and infer transmission patterns.

Whole-genome sequencing (WGS) approaches are gradually replacing spoligotyping and MIRU-VNTR in clinical and public health laboratories. TB has a small, single-chromosome genome of approximately 4.4 megabases (Mb) with low levels of homoplasy [10], rendering it amenable to WGS approaches [9]. With increasing access to WGS technology and the expanding volume of data in international databases such as the Tuberculosis Database [11] and the Relational Sequencing Tuberculosis Data Platform (ReSeqTB), laboratories worldwide may compare the single-nucleotide polymorphisms (SNPs) with those found in historical strains catalogued locally and elsewhere.

In Singapore, the use of WGS for public health surveillance began in 2014 as a grant-funded public health research project overseen by STEP and the Saw Swee Hock School of Public Health, National University of Singapore (SSHSPH-NUS). During the research phase, 499 retrospective MDR-TB and suspected outbreak samples of TB DNA were sent to Polaris in the Genome Institute of Singapore for sequencing followed by SSHSPH-NUS for bioinformatics analysis. The WGS results were utilized by STEP for cluster investigation and treatment of MDR-TB cases [12, 8].

Starting in 2019, the National Public Health Laboratory (NPHL) took over the TB WGS research programme as TB WGS transitioned to a full-fledged national public health surveillance programme supporting STEP [later renamed as the National TB Programme (NTBP)]. Located at the National Centre of Infectious Diseases, NPHL is the Ministry of Health’s public health diagnostics unit with purview over laboratory testing of pathogens of public health significance. As there are only two diagnostic mycobacteriology laboratories with biosafety level 3 (BSL-3) facilities in Singapore, the majority of the samples received by NPHL were selected retrospective samples from the Central Tuberculosis Laboratory (CTBL) at Singapore General Hospital. The selection criteria were based on suspected MDR-TB cases and cluster investigation cases. As sequencing and bioinformatics analytics capacity expanded, NPHL started accepting TB DNA samples on a prospective basis from CTBL as well as the National University Hospital Tuberculosis Laboratory (NUH TB Lab). Since November 2020, all culture-positive newly diagnosed cases of TB disease identified by CTBL and NUH TB Lab have mycobacterial genomic DNA extracted and forwarded to NPHL for WGS. NPHL reports all WGS results to the NTBP for case matching and further public health action.

The increasing availability of WGS data for TB cases resulted in MIRU-VNTR losing its relevance as a method of inferring relatedness to historical strains. The 24-loci MIRU-VNTR was phased out by CTBL in November 2020 [13].

## Operational workflow of tuberculosis WGS

As of January 2023, NPHL has sequenced 3808 TB samples since commencing TB WGS in 2019. Between 2019 and 2022, a total of 5309 new cases of TB were diagnosed among Singapore citizens and permanent residents.

Sequencing caseload has increased with the introduction in November 2020 of nationwide prospective screening, since which time 100 % of culture-positive samples from new cases of TB are sent for sequencing.

**Fig. 1.**
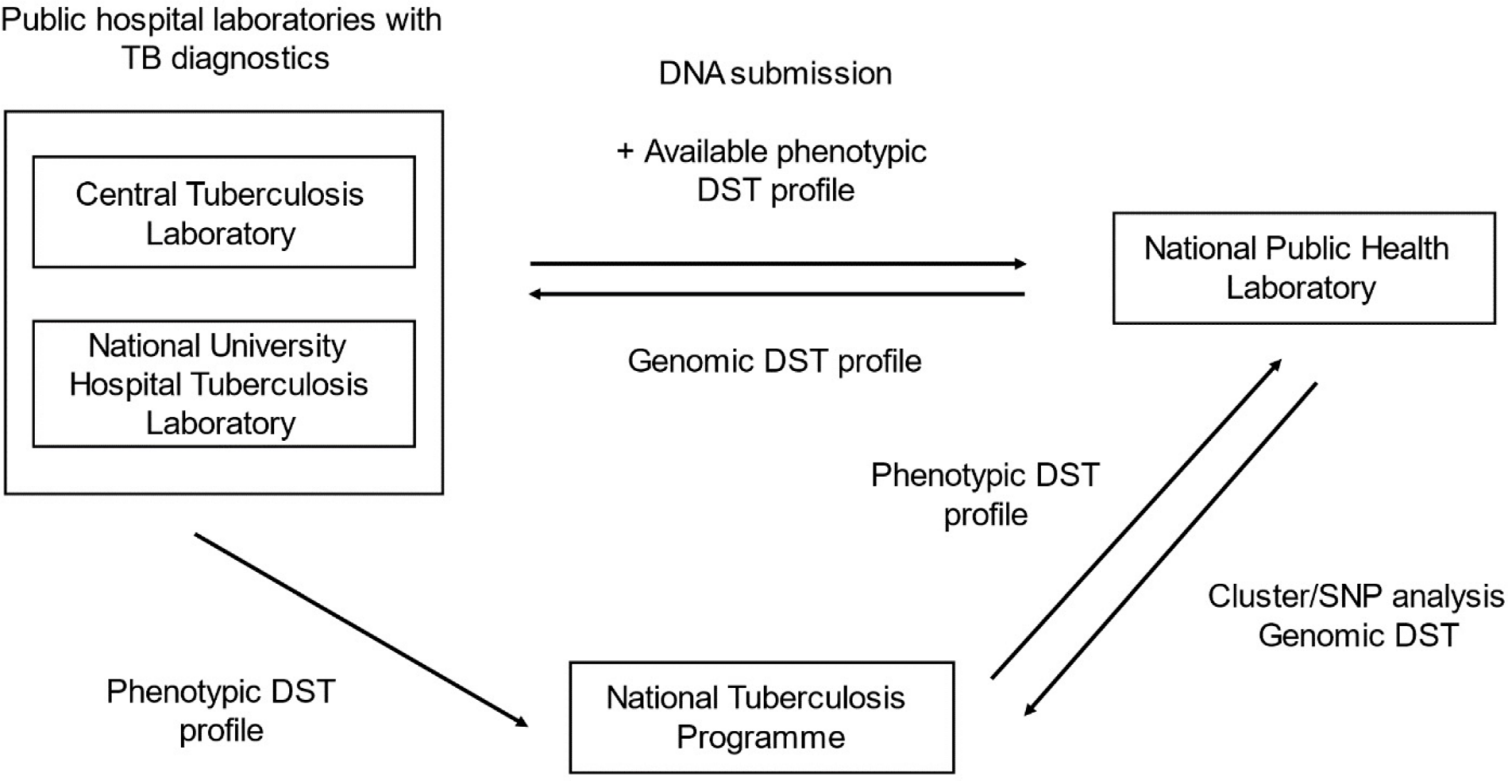
Operational workflow illustrating the role of NPHL’s WGS programme in the public health surveillance of TB in Singapore. DNA is submitted by two public hospital laboratories with phenotypic DST profiles. Following WGS analysis, genomic DST results are returned to these laboratories and NTBP. Genomic DST results complement phenotypic DST in informing clinical decisions on TB pharmacotherapy. Cluster analysis results are provided to NTBP.

Fig. 1 shows the operational workflow at NPHL. The two referring TB diagnostic laboratories extract genomic DNA (gDNA) from all culture-positive specimens belonging to newly diagnosed cases of TB. The concentration and purity of DNA are determined by Qubit fluorometry (Invitrogen, Waltham, MA, USA) and the 260/280 absorbance ratio. The referring laboratories send gDNA satisfying the minimum DNA concentration and purity requirements on a weekly basis to NPHL. Submitted gDNA must satisfy a minimum DNA concentration of 10 ng ml^−1^, and have a 260/280 ratio of between 1.6 and 2.4. Where available, phenotypic drug susceptibility testing (DST) results are also submitted by the originating laboratories to highlight drug-resistant and multidrug-resistant isolates.

Upon receipt of genomic DNA, NPHL performs WGS and bioinformatics analysis in batches, performing one to two WGS runs weekly. Prior to epidemiological and public health reporting to NTBP, NPHL informs clinicians of genomic DST results as soon as they are available, to avoid any delays for clinical intervention, if necessary. For isolates with discrepant phenotypic–genomic DST results, clinicians may consult NPHL and respective forwarding laboratories to discuss specific cases. Reporting of WGS results to NTBP usually takes ~2–4 weeks and follows laboratory-developed standardized formats for genomic DST and cluster analysis results, which is comparable to the few weeks required for phenotypic DST. Co-located in the National Centre for Infectious Diseases, NPHL and NTBP jointly organize weekly epidemiological meetings to encourage discussion and feedback on the data exchanged between the two public health units. Additionally, at quarterly to biannual intervals, NPHL issues aggregated genomic DST reports to the TB diagnostic laboratories, and coordinates periodic meetings among the laboratories for review of the DST correlation data. While individual WGS reports are not provided to the forwarding laboratories, the laboratories may inquire about specific cases on an ad-hoc basis.

## Technical workflow of tuberculosis WGS: phylogeny and genomic drug susceptibility testing pipelines

Starting with the Nextera XT DNA Library Preparation kit (Illumina, San Diego, CA, USA), each genomic DNA sample is tagmented, amplified with unique sequencing indexes and cleaned up using Ampure beads with size selection. The resulting individually tagged libraries are assessed for quality with a high-sensitivity DNA assay on an Agilent 2100 Bioanalyzer system for size distribution and average library sizes. Library concentrations are also measured via the Quant-iT dsDNA High-Sensitivity kit (Invitrogen). Library molarities are then calculated using average library sizes and measured DNA concentrations for manual normalization and pooling of 30–40 libraries into a single reaction. Pooled libraries are then loaded into the MiSeq Reagent kit v3 cartridge on the Illumina MiSeq platform (Illumina, San Diego, CA, USA) to generate 2×300 bp reads with an average processing time of 55 h.

Raw fastq files are transferred into NPHL’s high-performance computing workstation for bioinformatics analysis. Sequencing data are quality checked (QC) for Q30 scores, species purity and sequencing depth of >50×. Since the start of the programme, Q30 scores have been in the range of 60–80 %, with an average Q30 score of 70.4 %. Samples not meeting the minimum sequencing depth of >50×, found to be nontuberculous mycobacteria (NTMs), or contaminated with non-MTB reads are excluded from further downstream analysis. Sequences passing QC are analysed using two customizable bioinformatics WGS pipelines that have been adapted for in-house analysis, TBProfiler [14, 15] for drug resistance prediction and MTBseq [16] for clustering analysis. Briefly, paired-end reads are mapped to the H37Rv reference genome (NCBI accession number NC000962.3), variant called and annotated to generate a list of high-quality variants. TBProfiler also performs insertion and deletion (indel) calling using Delly software [17].

Drug resistance prediction mutations are reported with pre-assigned confidence scores from an in-house drug resistance confidence database. Currently, the database is not accredited to International Organization for Standardization (ISO) standards, but there are plans for ISO accreditation in the future. The drug resistance confidence database was built using three main references, the World Health Organization’s (WHO’s) 2018 drug resistance catalogue, the TBDB confidence database [11] and Miotto and colleagues’ standardized approach for reporting and interpretation of TB genotypic–phenotypic correlation data [18]. The confidence score for each drug resistance mutation is assigned based on three criteria:

1. Association of the mutation with phenotypic drug resistance, including frequency in drug-resistant isolates;
2. Amount and quality of evidence that the mutation is associated with drug resistance;
3. Distribution of minimum inhibitory concentrations for the drug in isolates possessing the mutation.

Our confidence database has recently been updated with the release of WHO’s 2021 drug resistance catalogue, which encompasses a wider variety of mutations with a more comprehensive scoring approach. NPHL has chosen to maintain its own database, instead of using an existing database such as the WHO’s 2021 catalogue, since the confidence score we assign is based on a holistic assessment of several references. The confidence scores assigned by WHO’s 2021 catalogue of mutations are based on their dataset of TB samples, which may differ from our local setting. For some rare mutations or drug-resistant mutations corresponding to newer compounds, WHO’s dataset may be too small for statistical significance to be reached and thus the confidence score needs to consider additional literature. Furthermore, we compare how the mutation fares in response to other more well-studied drugs with similar mechanisms of action.

NPHL follows three broad methodological principles in performing assessment of mutations:

1. For mutations lacking consensus or uncatalogued mutations, we prepare and evaluate an additional literature review alongside existing in-house phenotypic–genomic DST correlation data;
2. When we encounter rare mutations that are not well described in the references, we will search the literature to assess if there is a need to revise the initial scores we assigned to the mutation;
3. When mutations encountered are not associated with resistance, we do not remove mutations in its database; instead, we indicate that the mutation is not associated with resistance. This is because the literature may change over time and so the confidence score may be revised in future.

WGS clusters are reported using a 12 base pair (bp) threshold to the nearest cluster member. Newly detected clusters are assigned with a unique cluster identification number (ID). Isolates found to belong to previously reported clusters are assigned the same unique cluster ID. For clusters with four or more samples, phylogenetic analysis is performed with maximum-likelihood phylogenetic trees to hypothesize the possible evolutionary relationships between isolates in the same cluster. Isolates ≥12 bp to existing isolates are reported as ungrouped. Drug resistance variants are reported together with cluster analysis results in a standardized format to NTBP, who will distribute the reports to other relevant parties.

## Public health applications of tuberculosis WGS phylogeny

**Fig. 2.**
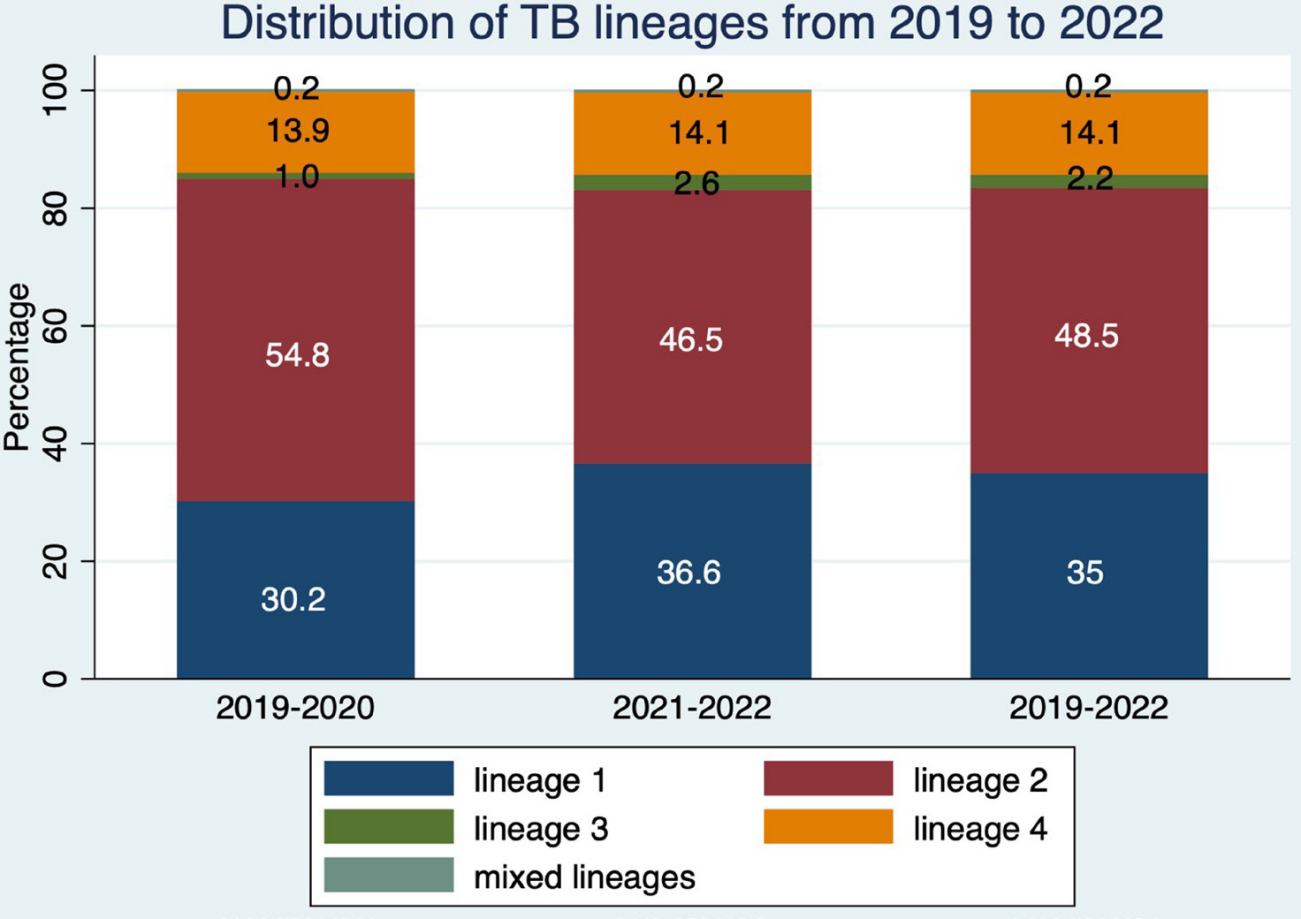
Distribution of TB lineages from 2019 to 2022. Most cases belong to lineages 1 and 2. The distribution of lineages sequenced before November 2020 may be subject to selection bias because the WGS programme in 2019–2020 had selectively sequenced retrospective MDR-TB samples and TB samples in MIRU-VNTR clusters. Samples from 2021 onward are more representative of actual current lineage distribution.

Lineage classification is useful for the monitoring of the major circulating human-associated lineages in Singapore. Fig. 2 shows the distribution of lineages in the sequenced TB samples between 2019 and 2022. Of the 3808 samples sequenced by NPHL between 2019 and 2022, 1847 samples (48 %) belonged to East-Asian lineage 2. The second and third most common lineages were Indo-Oceanic lineage 1 and lineage 4, contributing 1334 samples (35 %) and 535 samples (14 %), respectively.

WGS has also proven to be valuable as a tool for detecting transmission clusters beyond the usual contact tracing methods. Compared to spoligotyping and MIRU-VNTR, which inadvertently clusters closely related strains that do not have true epidemiological links, WGS has been shown to detect clusters with higher resolution and a lower false-positive rate. WGS may mitigate the tendency for standard genotyping methods to overestimate recent TB transmission among strains arising in settings with higher incidence rates [19].

The low specificity of spoligotyping and MIRU-VNTR is relevant to Singapore, a moderate-burden country situated in a geographical region with a moderate to high burden of TB disease. A molecular epidemiology study of 1612 non-duplicate TB isolates archived between 2006 and 2012 by CTBL found that although approximately 30 % of isolates clustered by combined spoligotyping and MIRU-VNTR, most of these clusters were false-positives as only a minority of clusters had demonstrable epidemiological links [20]. Cluster investigation based on spoligotyping and MIRU-VNTR results is likely to overestimate transmission due to the high prevalence of East-Asian lineage 2 in Singapore and in the region, a lineage for which clustering approaches based on spoligotyping and MIRU-VNTR have low discriminatory power [21].

The potential benefits of WGS for TB surveillance in Singapore were shown in a retrospective analysis of the 290 MDR-TB cases diagnosed in Singapore from 2006 to 2018 [12]. WGS-informed cluster investigation led to the evaluation of approximately one-third of the number of clusters and clustered patients identified by spoligotyping and MIRU-VNTR. Whereas spoligotyping and MIRU-VNTR identified 108 patients in 24 clusters, WGS analysis identified subgroups within these clusters, finding 43 patients clustered in 9 different clusters. One spoligotype–MIRU cluster of 12 cases was split into 3 separate WGS clusters, of which 1 WGS cluster was later proven to demonstrate epidemiological links, triggering additional contact investigation that uncovered an additional case. The two largest WGS clusters comprising 7 and 16 cases, respectively, had epidemiological links established for most individuals.

With universal and prospective nationwide implementation of a molecular genotyping method for all culture-positive newly diagnosed cases of TB, having a low false-positive rate results in a smaller search space for potential transmission chains, which saves on human resources and time.

Since the WGS programme was set up, NTBP has used the information to investigate several TB clusters. WGS has helped to guide public health actions in three main aspects: expansion of contact screening, triggering mass screening exercises and establishment of undetected social networks.

### Expansion of contact screening

WGS phylogeny results have been especially useful in justifying the expansion of contact screening efforts. The expanded contact screening resulted in the detection of clusters not evident on initial contact tracing.

In January 2021, two individuals attending a daycare centre were diagnosed with TB. Their visits to the daycare centre overlapped between July and September 2020. As part of routine contact screening, household members and clients were screened. The attack rates for the two index cases were 44.0 and 53.1 %. WGS revealed that these two index cases differed by only one SNP, suggesting transmission within the daycare centre. The confirmation of this link helped to support the need for expanded contact tracing and screening in the centre.

### Mass screening

WGS had also confirmed the presence of TB clusters within public housing blocks. A mass screening exercise was undertaken in response to a cluster of MDR-TB in a single 11-storey apartment block [8]. In a 4 year period, six residents were diagnosed with MDR-TB but none of them had any epidemiological links. TB screening was performed on 259 current or former residents. WGS of a total of 10 identified cases found that 8 of the 10 isolates were genetically identical and the remaining 2 isolates differed by only 1 SNP. If it were not for the WGS results, screening might not have been offered, and potentially infectious TB cases would not have been identified and treated.

Similarly, in October 2020, a cluster of four cases was detected within a public housing block. As the cases were living in different units, with no known epidemiological links apart from their residential address, this led to a mass screening exercise to screen the entire block.

### Establishment of social networks

In early 2021, a retrospective WGS analysis of current and historical tuberculosis cases revealed a large WGS cluster of 25 cases diagnosed between 2015 and 2020. Further epidemiological investigation revealed that some of these cases reported frequent exposure to a local betting centre with live broadcasts of horse racing [22]. Epidemiological investigation revealed that 10 cases had probable or possible links to the centre. The WGS cluster was made up of two distinct MIRU types and therefore MIRU-based clustering would not have identified this cluster.

This case study demonstrates how WGS can be an adjunct to routine contact tracing, by identifying epidemiological links in a social setting. The routine approach would not have identified casual contacts for screening. This also led to additional targeted screening for persons who had frequented the centre over the infectious period. WGS rationalized contact tracing efforts, facilitating direct questioning about places or activities common to other members of the WGS cluster.

## Implementation challenges of tuberculosis WGS phylogeny

*Mycobacterium tuberculosis* is estimated to have an average mutation rate of 0.5 SNPs per genome per year [23], although the mutation rate may be higher in settings with selective pressure of antituberculous therapy, such as in settings of high MDR-TB prevalence [24]. The slow rate of evolution of *M. tuberculosis* gives rise to relatively few SNPs in clinical isolates, which contributes to the challenge of phylogenetic reconstruction of the evolutionary relationships of clinical isolates [25], particularly in Singapore’s moderate-incidence setting.

A practical challenge in the bioinformatics analysis workflow we faced was the choice of SNP threshold to use for phylogenetic reconstruction. A single SNP threshold cannot possibly detect all epidemiologically linked cases in all contexts and all time frames, and therefore a threshold represents a trade-off. With a relaxed SNP threshold, more clusters may have to be investigated with a higher false-positive rate, contributing to increased staffing and funding required for contact tracing and epidemiological activities. Conversely, a more stringent SNP threshold may have a lower false-positive rate for WGS clusters being true clusters. However, a more stringent cut-off may result in missed WGS clusters and failure to act in situations where there may be transmission risks.

Different studies in the literature, mostly in low-incidence settings, have used varying SNP thresholds, typically 5 SNPs for detecting highly related clusters or clusters with recent transmission, and 12 SNPs for detecting potentially related clusters [26 9, 27, 28]. Assuming an average mutation rate of 0.5 SNPs per genome per year, these SNP thresholds correspond to transmission event detection windows of 10 and 24 years, respectively. From 2017 to 2019, a WGS-based surveillance system was piloted in all European Union/European Economic Area countries, using a SNP threshold of 5 or less to identify clusters with increased likelihood of recent transmission; analysis of 2218 rifampicin-resistant and MDR TB strains identified using a SNP threshold of 5 found 56 cross-border clusters with increased likelihood of recent transmission [29].

Since the establishment of the WGS programme, the upper threshold limit of 12 SNPs was used to define genetic clusters of isolates. However, we recently reviewed the SNP threshold by analysing the WGS clusters and epidemiological findings that had been investigated in 2020–2022. This review was performed because there are limited data in the literature to answer the question of whether a 12 SNP threshold may be too high in a moderate-incidence urban endemic setting such as ours. The outcome of the review was a downward adjustment in the SNP threshold to seven SNPs or fewer from January 2023 onwards for cluster detection. A tighter SNP threshold is thought to likely reduce the number of false-negative WGS clusters requiring public health investigation, while still maintaining similar sensitivity for cluster detection (manuscript in preparation). Had this tighter threshold been applied in 2020–2022, no clusters of public health importance would have been missed, and several false-positive clusters would not have required futile investigation.

## Clinical and public health applications of genomic DST (gDST)

Apart from phylogenetic analysis for purposes of lineage classification and cluster detection, gDST is the other major use case of WGS. The gDST pipeline predicts the drug resistance profile of a TB isolate by identifying mutations in core genes known to be associated with drug resistance. gDST prediction complements phenotypic DST (pDST) performed at the public hospital laboratories, the traditional, albeit imperfect, ‘gold standard’ for drug susceptibility testing. For routine samples, phenotypic DST is based on mycobacteria growth indicator tube (MGIT) drug susceptibility testing.

Since most TB cases in Singapore are pan-sensitive, it could be argued that genomic DST could potentially replace phenotypic DST for presumptive pan-sensitive cases, should there be cultures available, and assuming a turnaround time (TAT) for genomic DST returns equivalent to, or faster than, phenotypic DST. Even in such an approach where genomic DST is first line, phenotypic DST should still be performed alongside genomic DST for suspected drug-resistant cases (such as presumptive rifampicin-resistant cases and/or MDR), delayed culture conversion, or relapse. However, in Singapore TB WGS has not so far been accredited as an *in vitro* diagnostics test for routine clinical reporting. Furthermore, the test is performed in batches for the primary purpose of public health surveillance, and therefore the TAT would not be rapid enough for clinical workflows, especially considering the moderate to high year-round incidence of TB disease in Singapore. Therefore, pDST remains the first line for DST, with gDST serving a complementary role. In the future, gDST may become the first-line DST method if the following criteria are fulfilled:

1. Authorization of gDST as an *in vitro* diagnostics test for routine clinical reporting;
2. Efficiency and cost-effectiveness: the TAT should be as fast as, if not faster than, pDST;
3. Validation of gDST predictions in the local context of TB genetic epidemiology in Singapore: assuming that the incidence of drug resistance in Singapore remains low, if or when gDST is approved as an *in vitro* diagnostics test, gDST could replace pDST for first-line testing. By virtue of its high negative predictive value, if no genotypic resistance is detected, the probability of pan-sensitivity would be high;
4. A framework for appropriate case selection, where samples with a high pre-test probability for resistance could be prioritized for gDST: further statistical and epidemiological analyses could be performed to identify patient-specific variables that increase risk of resistance, for example countries of origin, previous treatment history, or non-compliance to treatment. Samples arising from these ‘high-risk’ patients, as well as isolates with an initial indication of rifampicin resistance (e.g. on GeneXpert), could be prioritized for gDST.

gDST prediction results are reported for all first-line oral anti-tuberculous drugs (isoniazid, rifampicin, ethambutol and pyrazinamide), streptomycin and fluoroquinolones. Since isolates known to be resistant to rifampicin are treated as presumptive MDR-TB cases, resistance predictions for all other TB drugs available for testing, including streptomycin and the fluoroquinolones, are reported for such isolates. All newly reported genomic DST results are catalogued in NPHL’s in-house database for future reference.

**Fig. 3.**
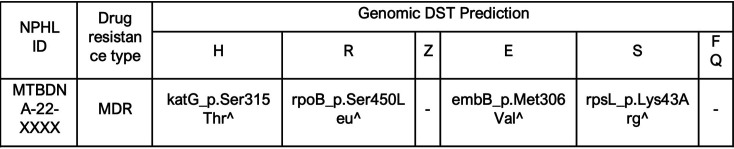
Example of genomic DST prediction report. H, isoniazid; R, rifampicin; Z, pyrazinamide; E, ethambutol; S, streptomycin; FQ, fluoroquinolones. A symbol abbreviation is used to indicate confidence scoring for each detected drug mutation associated with phenotypic drug resistance. ^, high confidence; *, low confidence; ~, indeterminate confidence. In this example, all detected drug mutations for isoniazid, rifampicin, ethambutol and streptomycin have been assigned high (^) confidence scores.

Fig. 3 shows a snippet of a genomic DST prediction report issued for a single sample. For each detected drug mutation associated with phenotypic drug resistance, a confidence score of high, low, or indeterminate is assigned based on NPHL’s in-house database. Detailed sample reports are available in the Supplementary Material.

Resistance mutation databases are dynamic records of mutations that are updated from time to time. Databases do not exhaustively list all the possible genetic drivers of resistance. Uncharacterized or novel mutations may not be catalogued. Furthermore, genomic DST is unable to resolve the confounding effects of mixed infections or heteroresistance.

Genomic DST has a high degree of concordance with phenotypic DST (Table 1). The highest level of concordance (accuracy score) is seen with rifampicin, with 99.6 % of samples with paired genomic and phenotypic DST showing concordant genomic and phenotypic rifampicin resistance predictions.

**Table 1.** Calculated performance metrics for resistance detection by genomic DST performed at NPHL, with respect to the reference standard of phenotypic DST performed at the clinical mycobacteriology laboratories

Anti-TB drug	no. of samples with paired data	no. of concordant samples	Concordance rate (accuracy) (%)	Sensitivity (%)	Specificity (%)	Positive predictive value (%)	Negative predictive value (%)
R	2996	2983	99.6	98.4	99.6	83.6	99.97
H	2994	2972	99.3	94.8	99.6	94.8	99.6
E	2998	2959	98.7	92.9	98.7	25.5	99.97
Z	2138	2120	99.2	65.2	99.9	93.8	99.2

Positive, drug-resistant; negative, drug-sensitive; R, rifampicin; H, isoniazid; E, ethambutol; Z, pyrazinamide.

Across all four first-line antituberculous drugs, negative predictive values, which exceed 99 % for all four drugs, are consistently higher than positive predictive values. The gap between negative predictive value and positive predictive value is especially striking with ethambutol. Among the 51 samples with genomically predicted ethambutol resistance, only 13/51 (25.5 %) had confirmed phenotypic ethambutol resistance. The high number of false positives on ethambutol genomic DST suggests that the mutations relied on to predict ethambutol resistance are poorly associated with phenotypic resistance on MGIT testing.

Furthermore, across all four first-line drugs, the sensitivity of genomic DST is consistently poorer than specificity. For pyrazinamide, the poor sensitivity of 65.2 % indicates that in over a third of phenotypically resistant samples, no resistance-conferring mutations were identified. The poor concordance between genomic and phenotypic DST demonstrates the need for further characterization of the genetic determinants of phenotypic resistance.

While genomic DST is a promising methodology that could inform clinical therapeutics, the challenges of low positive predictive values and suboptimal sensitivities limit its effectiveness. Interpretation of the performance of genomic DST in pyrazinamide susceptibility testing may be further complicated by questions of the reliability of the comparator test, MGIT [30]. Additional investigation into genomic–phenotypic correlation is likely necessary for pyrazinamide susceptibility testing, as well as for the second-line drugs, for which fewer data are currently available.

## Concluding remarks

WGS in Singapore started out as a research project that retrospectively sequenced selected MDR-TB cases and suspected cluster cases for drug resistance predictions and cluster detection analysis. With increasing recognition of the utility of WGS and availability of funding, the programme was transitioned to a universal prospective programme led by NPHL, which gradually took over the wet-lab and dry-lab functions of the programme in 2019 and 2020, respectively. Currently, the WGS programme prospectively sequences all culture-positive newly diagnosed cases of TB in the country, achieving the main use cases of phylogenetic surveillance and genomic drug susceptibility testing. Phenotypic drug susceptibility testing continues to be performed at the two clinical mycobacteriology laboratories. The nationwide implementation of WGS for all TB isolates in Singapore is a useful case study for successful implementation of WGS in a high-resource, moderate-incidence jurisdiction, and an illustration of how WGS provides the information required to track transmissions with high resolution and to individualize TB treatment, especially for MDR-TB cases.

There have been numerous reports of implementation of WGS in settings with differing resource levels and incidence rates. For example, WGS has been implemented in low-incidence jurisdictions such as the UK[31], Italy [32], and the Australian state of Victoria [26], as well as moderate to high-incidence jurisdictions such as the Kyrgyz Republic [33]. If financial and labour resources are not a constraining factor for a national public health laboratory, then an implementation of nationwide prospective WGS paralleling NPHL’s approach is likely to address many public health applications in inferring cluster relationships and informing the treatment of MDR-TB cases. However, laboratories in high-incidence settings with resource constraints may encounter barriers to implementation of universal WGS. Funding constraints may limit WGS to a selective, targeted sampling approach, especially if the perceived utility of WGS has not been proven in a local context. Furthermore, it takes time to train personnel, build up partnerships with public health agencies and programmes involved in TB control, and establish workflows with treating clinicians and diagnostic laboratories. Laboratories in resource-constrained high-incidence settings that may wish to establish a WGS service may find NPHL’s experiences with transitioning from selective WGS to universal WGS to be instructive. These laboratories may develop a WGS programme gradually, starting with a pilot for selected samples, focusing on drug resistance surveillance for MDR-TB strains and phylogenetic analysis of selected ‘hot spot’ areas [34]. If successful, the WGS pilot would demonstrate the utility of WGS for informing public health actions, which may be useful in justifying expansion of the service to policymakers.

As people flows normalize to pre-coronavirus disease 2019 (COVID-19) pandemic levels, Singapore continues to be exposed to the risks of trans-national TB spread. For WGS to be an effective tool in curbing trans-national spread, international WGS data must be made accessible by public health professionals. The barrier is that such data are not available currently. Various solutions to this problem may be implemented. These solutions require political will, funding and labour resources on the national and international levels. Countries with moderate to high burden with pre-existing WGS programmes may consider expanding their services and sample load to improve representativeness. Countries without a WGS programme may consider starting one, perhaps with national and international funding designed to increase access to WGS technology and expertise. Furthermore, the WGS data should be shared between countries, following the example of the GISAID database that exists for severe acute respiratory syndrome coronavirus 2 (SARS-CoV-2). Quality control, analysis and interpretation aspects of WGS should be standardized to support cross-border collaboration [35]. We suggest that international standards for WGS protocols and reporting may need to be developed, like those available for SARS-CoV-2 [36]. On a regional level, workshops and seminars could be organized to harmonize practices among public health laboratory professionals from different countries, facilitate knowledge sharing and collaboration, and promote the routine use of WGS as a surveillance tool in the control of TB.

Globally, the priority for TB molecular epidemiology is to increase laboratory funding, improve the cost-effectiveness of sequencing equipment and consumables, and create training pathways to equip laboratory professionals with WGS wet-lab techniques and bioinformatics analysis methods. Access to targeted or universal WGS must be democratized so that low-resource, high-burden jurisdictions also have the capacity to perform targeted or universal WGS for TB strains on a prospective basis. With increasing accessibility to WGS technology, national public health laboratories should seek to implement WGS for prospective cluster detection purposes and genomic drug susceptibility testing and expand data exchange programmes with other national public health laboratories. International collaboration between public health laboratories and health ministries will allow early outbreak detection and facilitate the interruption of transmission dynamics both within and across national and regional borders. TB is an ancient disease requiring modern technology and methods in response, not just in clinical diagnostics and therapeutics, but also in surveillance. To improve our chances of eradicating TB in the 21st century, laboratory and public health professionals must effectively and fully utilize WGS as a method of molecular surveillance.

## Supplementary Data

Supplementary material 1

Supplementary material 2

Supplementary material 3
