# Supplementary material for: Implementation of national whole-genome sequencing of Mycobacterium tuberculosis, National Public Health Laboratory, Singapore, 2019—2022
Source: Microb Genom. 2023 Nov 27;9(11):001139. doi: 10.1099/mgen.0.001139 (PMC10711301; doi:10.1099/mgen.0.001139)
Supplement: Supplementary material 1 [file mgen-9-1139-s001.pdf]

Report for TB WGS Analysis / Batch XX

Submitting Lab Number	Name	NPHL ID	Depth	Lineage	Genomic DST Prediction								Group	Cluster	Additional Remarks, if any
					DR type	H	R	Z	E	S	FQ	Others			
123456789	ABC	MTBDNA-23-XXXX	78	lineage4.4.2	Pansens	-	-	-	-	-	-	-	123	123-1	
234567891	BCA	MTBDNA-23-XXXX	58	lineage2.2.1	DR	-	-	pncA_p.Ile90Ser^	-	-	-	-	ungrouped		
345678912	CAB	MTBDNA-23-XXXX	87	lineage2.2.1	MDR	katG_p.Ser315Thr^	rpoB_p.His445Arg^	-	embB_p.Met306Ile^	rpsL_p.Lys43Arg^	-	-	ungrouped		
456789123	ACB	MTBDNA-23-XXXX	61	lineage1.1.2	Pansens	-	-	-	-	-	-	-	20	20-2	
567891234	BAC	MTBDNA-23-XXXX	61	lineage4.5	Pansens	-	-	-	-	-	-	-	ungrouped		
678912345	CBA	MTBDNA-23-XXXX	84	lineage3	DR	katG_p.Ser315Thr^	-	-	-	rpsL_p.Lys43Arg^	-	-	ungrouped		

Symbol	Confidence Scoring for detected drug mutations associated with phenotypic drug resistance
^	High
*	Low
~	Indeterminate

-SAMPLE ONLY-

Points to Note:

1. Reported drug resistance mutations are taken from the TBDB mutation library (https://github.com/jodyphelan/tbdb), which may be subject to changes over time with new literature.
2. Phenotypic DST is still necessary to complement genomic DST prediction as WGS cannot detect novel, uncharacterized mutations.
3. Confidence scoring for genomic DST prediction are subject to NPHL's interpretation based on the following references:

World Health Organization. (2021). Catalogue of mutations in Mycobacterium tuberculosis complex and their association with drug resistance. Geneva: World Health Organization; 2021. Licence: CC BY-NC-SA 3.0 IGO.

World Health Organization. (2018). The use of next-generation sequencing technologies for the detection of mutations associated with drug resistance in Mycobacterium tuberculosis complex: technical guide. World Health Organization. https://apps.who.int/iris/handle/10665/274443. License: CC BY-NC-SA 3.0 IGO

Miotto P, Tessema B, Tagliani E, et al. A standardised method for interpreting the association between mutations and phenotypic drug resistance in Mycobacterium tuberculosis. The European respiratory journal 2017; 50(6).

TBDB confidence database. https://github.com/jodyphelan/tbdb.

4. Genomic DST predictions for all samples are reported only for the listed drugs in this report - Isoniazid (H), Rifampicin (R), Pyrazinamide (Z), Ethambutol (E), Streptomycin (S) and Fluoroquinolones (FQ). Full genomic DST prediction will be reported only for known Rif-R isolates. This can also be provided for isolates of interest upon request.
5. Drug resistance predictions made via WGS have not been validated for clinical use.

Supplemental Figure 1: Example of a genomic DST prediction and cluster analysis report for NTBP. Drug abbreviations: H = isoniazid, R = rifampicin, Z = pyrazinamide, E = ethambutol. S = streptomycin, FQ = fluoroquinolones. A symbol abbreviation is used to indicate confidence scoring for each detected drug mutation associated with phenotypic drug resistance. ^ indicates High confidence, * indicates Low confidence, and ~ indicates Indeterminate confidence.

Supplemental Table 1: Description for column headers in the TB WGS analysis report for NTBP

<table>
<tr><th>Header</th><th colspan="2">Description</th></tr>
<tr><td>Submitting Lab number</td><td colspan="2">Unique identifier provided by submitting lab</td></tr>
<tr><td>Name</td><td colspan="2">Patient initials</td></tr>
<tr><td>NPHL ID</td><td colspan="2">NPHL unique identifier assigned to submitted sample</td></tr>
<tr><td>Depth</td><td colspan="2">Mean Coverage Depth</td></tr>
<tr><td>Lineage</td><td colspan="2">TB lineages</td></tr>
<tr><td rowspan="4">DR type</td><td>Pansens</td><td>Pan-sensitive</td></tr>
<tr><td>DR</td><td>Drug mutation</td></tr>
<tr><td>MDR</td><td>At least RIF-R and INH-R</td></tr>
<tr><td>XDR</td><td>At least RIF-R, INH-R, FQ-R and AI-R</td></tr>
<tr><td rowspan="17">Genomic DST predictions</td><td>H</td><td>Isoniazid (INH)</td></tr>
<tr><td>R</td><td>Rifampicin (RIF)</td></tr>
<tr><td>Z</td><td>Pyrazinamide (PZA)</td></tr>
<tr><td>E</td><td>Ethambutol (EMB)</td></tr>
<tr><td>S</td><td>Streptomycin (STM)</td></tr>
<tr><td>FQ</td><td>Fluoroquinolones (FQ)</td></tr>
<tr><td rowspan="11">Others</td><td>Aminoglycoside injectables (AI)</td></tr>
<tr><td>Amikacin (AMK)</td></tr>
<tr><td>Kanamycin (KAN)</td></tr>
<tr><td>Capreomycin (CAP)</td></tr>
<tr><td>Ethionamide (ETH)</td></tr>
<tr><td>Para-aminosalicylic acid (PAS)</td></tr>
<tr><td>Cycloserine (CYS)</td></tr>
<tr><td>Linezolid (LZD)</td></tr>
<tr><td>Bedaquiline (BDQ)</td></tr>
<tr><td>Clofazimine (CFZ)</td></tr>
<tr><td>Delamanid (DLM)</td></tr>
<tr><td>Group</td><td>NPHL assigned WGS group number</td><td>Members in WGS groups with less than 3 isolates are recorded under 'Additional remarks'. For WGS groups with more than 3 isolates, phylogenetic trees are provided.</td></tr>
<tr><td>Cluster</td><td colspan="2">WGS sub-group number</td></tr>
<tr><td>Additional remarks</td><td colspan="2">Additional remarks such as mixed lineages, mapped reads, and additional DR analysis if any.</td></tr>
</table>

National Centre for Infectious Diseases

Bacteriology

Title: Laboratory Report on *Mycobacterium tuberculosis* Genomic DST Prediction
Date: DD/MM/YYYY
Sender's Institution:
Sender's Department:

S/N	NPHL Accession Number	Submitting Lab Number	Sample Received Date	Summary of Genomic DST Prediction								Remarks
				DR type	H	R	Z	E	S	FQ	Others	
1	MTBDNA-23-XXXX	123456789	1/1/2023	Pansens	-	-	-	-	-	-		
2	MTBDNA-23-XXXX	234567891	1/1/2023	DR	fabG1_c.-15C>T^	-	-	-	-	-		
3	MTBDNA-23-XXXX	345678912	1/1/2023	MDR	katG_p.Ser315Thr^	rpoB_p.Ser450Leu^	-	-	rpsL_p.Lys43Arg^	-		
4	MTBDNA-23-XXXX	456789123	1/1/2023	XDR	katG_p.Ser315Thr^	rpoB_p.Ser450Leu^	-	-	-	gyrA_p.Asp94Tyr^	Kanamycin: eis_c.-12C>T^	

Reported by:
Name of Staff

Designation
5/9/2023 10:13 AM

Remarks:
1. Confidence scoring for predicted drug mutations associated with phenotypic drug resistance is indicated as high (^), low (*) and indeterminate (~).
2. Reported drug resistance mutations are taken from the TBDB mutation library (https://github.com/jodyphelan/tbdb), which may be subject to changes over time with new literature.
3. Phenotypic DST is still necessary to complement genomic DST prediction as WGS cannot detect novel, uncharacterized mutations.
4. Confidence scoring for genomic DST prediction is subject to NPHL's interpretation based on the following references:

- World Health Organization. (2021). Catalogue of mutations in Mycobacterium tuberculosis complex and their association with drug resistance. Geneva: World Health Organization; 2021. Licence: CC BY-NC-SA 3.0 IGO.
- World Health Organization. (2018). The use of next-generation sequencing technologies for the detection of mutations associated with drug resistance in Mycobacterium tuberculosis complex: technical guide. World Health Organization. https://apps.who.int/iris/handle/10665/274443. License: CC BY-NC-SA 3.0 IGO
- Miotto P, Tessema B, Tagliani E, et al. A standardised method for interpreting the association between mutations and phenotypic drug resistance in Mycobacterium tuberculosis. The European respiratory journal 2017; 50(6).
- TBDB confidence database. https://github.com/jodyphelan/tbdb.

5. Genomic DST predictions for all samples are reported only for the listed drugs in this report - Isoniazid (H), Rifampicin (R), Pyrazinamide (Z), Ethambutol (E), Streptomycin (S) and Fluoroquinolones (FQ). Full genomic DST prediction will be reported only for known Rif-R isolates. This can also be provided for isolates of interest upon request.

6. Drug resistance predictions made via WGS have not been validated for clinical use.

SAMPLE ONLY -

National Public Health Laboratory@NCID
Block G, Level 13
16 Jalan Tan Tock Seng
Singapore 308442
Tel: 6357 7341 / 7343

Name of Staff
Director, NPHL

Name of Staff, PhD
Designation

Supplemental Figure 2: Example of a genomic DST prediction report. Drug abbreviations: H = isoniazid, R = rifampicin, Z = pyrazinamide, E = ethambutol. S = streptomycin, FQ = fluoroquinolones. A symbol abbreviation is used to indicate confidence scoring for each detected drug mutation associated with phenotypic drug resistance. ‘^’ indicates High confidence, ‘*’ indicates Low confidence, and ‘~’ indicates Indeterminate confidence.
